# Prokaryotic Life Associated with Coal-Fire Gas Vents Revealed by Metagenomics

**DOI:** 10.3390/biology12050723

**Published:** 2023-05-15

**Authors:** Vitaly V. Kadnikov, Andrey V. Mardanov, Alexey V. Beletsky, Olga V. Karnachuk, Nikolai V. Ravin

**Affiliations:** 1 Institute of Bioengineering, Research Center of Biotechnology of the Russian Academy of Sciences, 119071 Moscow, Russia; 2 Laboratory of Biochemistry and Molecular Biology, Tomsk State University, 634050 Tomsk, Russia

**Keywords:** thermophiles, metagenome, microbial community, *Firmicutes*, coal gases, hydrogenotrophy, carboxydotrophy, DTU015

## Abstract

**Simple Summary:**

The natural combustion of underground coal deposits leads to the release of large quantities of gases which contain molecular hydrogen and carbon monoxide. In places where hot coal gases are released to the surface, specific communities of thermophilic microorganisms develop. Unlike well-characterized geothermal ecosystems, the thermophiles associated with coal-fire gas vents are largely unexplored. In this work, using molecular genetic methods, we studied the composition and functional potential of bacterial and archaeal communities in the near-surface ground layer of an open quarry in Eastern Siberia, heated by an underground coal fire that began a few decades ago. The communities were dominated by only a few groups of bacteria of the phylum *Firmicutes*. Genome analysis predicted that they might obtain energy from the oxidation of hydrogen and carbon monoxide in coal gases. All these species were predicted to be spore-forming. Interestingly, closely related bacteria were found in various thermal environments, including burning coal seams, at distances of thousands of kilometers from the studied site. Spores of thermophilic *Firmicutes* can probably spread over long distances, which allows these microorganisms to colonize these thermal ecological niches.

**Abstract:**

The natural combustion of underground coal seams leads to the formation of gas, which contains molecular hydrogen and carbon monoxide. In places where hot coal gases are released to the surface, specific thermal ecosystems are formed. Here, 16S rRNA gene profiling and shotgun metagenome sequencing were employed to characterize the taxonomic diversity and genetic potential of prokaryotic communities of the near-surface ground layer near hot gas vents in an open quarry heated by a subsurface coal fire. The communities were dominated by only a few groups of spore-forming *Firmicutes*, namely the aerobic heterotroph *Candidatus* Carbobacillus altaicus, the aerobic chemolitoautotrophs *Kyrpidia tusciae* and *Hydrogenibacillus schlegelii*, and the anaerobic chemolithoautotroph *Brockia lithotrophica*. Genome analysis predicted that these species can obtain energy from the oxidation of hydrogen and/or carbon monoxide in coal gases. We assembled the first complete closed genome of a member of uncultured class-level division DTU015 in the phylum *Firmicutes*. This bacterium, ‘*Candidatus* Fermentithermobacillus carboniphilus’ Bu02, was predicted to be rod-shaped and capable of flagellar motility and sporulation. Genome analysis showed the absence of aerobic and anaerobic respiration and suggested chemoheterotrophic lifestyle with the ability to ferment peptides, amino acids, *N*-acetylglucosamine, and tricarboxylic acid cycle intermediates. Bu02 bacterium probably plays the role of a scavenger, performing the fermentation of organics formed by autotrophic *Firmicutes* supported by coal gases. A comparative genome analysis of the DTU015 division revealed that most of its members have a similar lifestyle.

## 1. Introduction

The long-term natural combustion of coal deposits leads to the formation of thermal ecological niches in which specific microbial communities can develop. Usually the combustion of coal leads to the release of a large amount of gases formed during the incomplete oxidation of carbon in the presence of water (reactions 3C + O_2_ + H_2_O > H_2_ + 3CO and CO + H_2_O > CO_2_ + H_2_). In addition to CO_2_, coal gases mainly contain hydrogen, CO, and gaseous hydrocarbons, i.e., their composition corresponds to the synthesis gas obtained in the course of artificial coal gasification [1,2]. These gases may also contain hydrogen sulfide, sulfur oxides, benzene, xylene, and aliphatic and halogenated compounds [2,3]. Sulfur and other elements present in coal deposits can be carried to the surface by gas flow, resulting in the contamination of surrounding areas [4]. In places where hot gases from coal combustion come to the surface, local extreme ecosystems can form, which are characterized by elevated temperatures (>50 °C).

The phenomenon of the underground combustion of coal seams is relatively common in nature and occurs in Australia, Germany, the USA (Pennsylvania), China, Russia, India, and other countries [1]. Underground fires can last for centuries, such as the coal seam at Dudweiler in Germany, burning since 1668, and the Burning Mountain in Australia, burning for about 6000 years. The temperature of the gas escaping to the surface depends on the temperature and the depth of the fire site and usually ranges from 50 to 800 °C. Local extreme ecosystems, characterized by high temperatures (>50 °C) and the presence of toxic substances, can form near coal-fire gas vents [5]. High-energy compounds in coal gases, such as hydrogen and CO, can be used by microorganisms to enable the development of specific communities of thermophiles. However, the composition and ecology of microbial communities in such environments and the genetic potential of these microorganisms are poorly known.

The first studies of microbial communities in such habitats were carried out by 16S rRNA gene profiling in Centralia in Pennsylvania, USA, in an area of underground coal mine combustion [5,6]. The same object was recently studied by metagenomic methods, and it was shown that bacteria with smaller cell and genome sizes were more abundant in soils with a higher temperature [7]. The composition of soil microbial communities around hot coal gas vents in Xinjiang, China was studied using T-RFLP analysis and 16S rRNA gene sequencing [3]. Among the dominant groups of microorganisms, representatives of the phyla *Firmicutes, Proteobacteria*, *Acidobacteriota, Bacteroidota*, *Planctomycetes*, and *Actinobacteriota* were detected. Metagenomic analysis of coal-bearing rocks and bituminous substances in an open coal quarry heated by a subsurface fire in the Altai Mountains (Russia) revealed the dominance of a few species of thermophilic *Firmicutes* that can gain energy from the oxidation of molecular hydrogen [8]. On the contrary, a microbial community in an area of coal-fire gas vents in the Kuznetsk Coal Basin was dominated by *Ktedonobacteria* (the phylum *Chloroflexi*), known to be capable of oxidizing hydrogen and carbon monoxide, while thermophilic hydrogenotrophic *Firmicutes* formed a minor part of the community [9]. Another site in this region mostly harbored bacteria of the genus *Thermus* and hydrogenotrophic *Firmicutes* [10]. These studies have shown that specific lineages of hydrogenotrophic thermophilic *Firmicutes* are ubiquitous in microbial communities associated with coal gas vents but do not always dominate them.

In this work, we analyzed the prokaryotic communities associated with the underground combustion of brown coal at a depleted coal deposit in Eastern Siberia (Republic of Buryatia, Russia). We report data on the composition of microbial communities obtained by the 16S rRNA gene profiling and sequencing of ground metagenome in the area where the hot gases escape to the surface. We assembled high-quality metagenome-assembled genomes (MAGs) of most members of the community, which allowed us to accurately reconstruct the metabolic pathways of microorganisms and identify the main microbial processes of carbon and sulfur cycling. One of the MAGs represented the first complete closed genome of a member of the candidate uncultured class-level division DTU015 of the phylum *Firmicutes*. Genome data were used for the reconstruction of the metabolic potential of the DTU015 bacterium, the prediction of its ecological roles, and the comparative genome analysis of this lineage.

## 2. Materials and Methods

### 2.1. Sampling, Field Measurements, Mineralogical Analyses, and DNA Isolation

**Figure 1:**
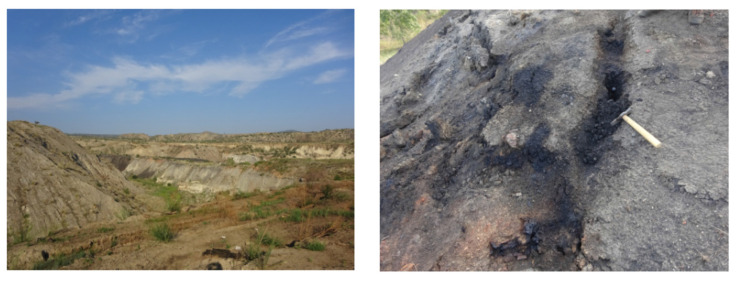
General view of the area where coal combustion occurs.

Samples of finely dispersed coal-bearing rocks in the area of an underground coal fire were taken from an abandoned coal pit near the city of Gusinoozersk in Eastern Siberia (Selenginsky district, Republic of Buryatia, Russia, coordinates 51.195168, 106.491027). At the time of sampling, signs of an underground coal fire were visible as the intense heating of the soil and rocks and the rise of vapor clouds (Figure 1). All samples were kept before analysis for 3–4 days at + 4 °C. DNA samples were isolated using the MO BIO Power Soil DNA Isolation Kit (Qiagen, Valencia, CA, USA) following the recommendations of the manufacturers. The sample with added lysis buffer was vigorously shaken twice in a Precellys Evolution Touch homogenizer (Bertin Technologies, Montigny-le-Bretonneux, France) at a speed of 4500 rpm for 25 s.

Scanning electron microscopy was used to characterize the content of the main elements. Microprobes were examined using a Philips SEM 515 microscope (Philips, Eindhoven, The Netherlands) with energy dispersive spectrometry as previously described [11]. X-ray diffraction (XRD) analysis was performed with a XRD-6000 instrument (Shimadzu Corporation, Kyoto, Japan).

### 2.2. 16S rRNA Gene Profiling

The universal primers 341F (5’-CCTAYGGGDBGCWSCAG-3′) and 806R (5′-GGACTACNVGGGTHTCTAAT-3′) [12] were used for the PCR amplification of V3-V6 variable regions of the 16S rDNA gene of archaea and bacteria. The libraries were indexed using the Nextera XT Index Kit v.2 (Illumina, San Diego, CA, USA) and sequenced on the MiSeq platform in a paired read format (2 × 300 nt). Paired reads were merged using FLASH v.1.2.11 [13]. Low-quality and chimeric sequences, as well as singletons, were filtered using the Usearch program [14]. The remaining sequences were binned based on 97% sequence similarity to designate the unique operational taxonomic units (OTUs) using Usearch [14].

The taxonomic identification of OTUs was performed by searches against the SILVA v.132 rRNA sequence database using the VSEARCH sintax algorithm [15].

### 2.3. Metagenome Sequencing, Assembly of Contigs, and Binning of MAGs

Metagenomic DNA from sample Bu22-1 was sheered to a fragment size of ~400 bp and used to prepare the TruSeq DNA library. The shotgun library was sequenced in paired-end mode (2  ×  150 bp) on a HiSeq 2500 instrument; a total of 109,964,469 read pairs were obtained. Sequencing adapters were removed with Cutadapt v.1.8.3 [16]. Sickle v.1.33 (https://github.com/najoshi/sickle (accessed on 20 March 2022)) was used for the trimming of low-quality reads (Q < 30). Trimmed reads were merged using FLASH v.1.2.11 [13].

In addition, metagenomic DNA was sequenced on a MinION system (Oxford Nanopore, Oxford, UK) using the 1D Genomic DNA by ligation protocol and kit (SQK-LSK108). Sequencing this library in an R9.4 flow cell (FLO-MIN106) using MinION instrument generated 8,444,927reads with a total length of 4.88 Gbp.

The obtained Illumina reads (about 24.0 Gbp) and Nanopore reads were *de novo* assembled into contigs using metaSPAdes hybrid assembler v.3.13.0 [17].

Contigs longer than 1500 bp were binned into MAGs using MetaBAT v.2.12.1 [18]. To improve MAG assembly, MinION reads were mapped to MAGs with BWA v.0.7.15 [19]. Then, the Npscarf v.1.0 program [20] was used to scaffold the contigs and to fill gaps between the scaffolded contigs using Illumina consensus sequences from the metaSPAdes assembly graph.

In addition, MinION reads were *de novo* assembled using Flye v. 2.7 [21]. Sequences of the obtained contigs were polished using Pilon v.1.2.2 [22] with two iterations of Illumina reads mapping back to the assembled sequences using Bowtie 2 [23]. The MinION contigs were binned into MAGs using MetaBAT v.2.12.1 [18].

### 2.4. Genome Annotation and Analysis

MAG completeness and possible contamination were assessed using the CheckM v.1.05 [24] lineage-specific approach. MAGs were identified according to the genome-based taxonomic system using the Genome Taxonomy Database Toolkit (GTDB-Tk) v.0.3.2 and Genome Taxonomy database (GTDB), revease R95 [25].

Based on the assembly statistics, completeness, and contamination values, four MAGs (Bu05, Bu31, Bu66, and Bu77) were taken from the Flye assembly; for other MAGs, metaSPAdes results were further used.

Gene prediction and annotation were performed using the NCBI Prokaryotic Genome Annotation Pipeline [26] or RAST server 2.0 [27]. To predict N-terminal signal peptides in proteins, the Signal P v. 5.0 program was used. Transmembrane domains were predicted using the TMHMM v.2.0 server (http://www.cbs.dtu.dk/services/TMHMM/ (accessed on 20 March 2022)).

Transporters were identified by searches against the transporter classification database [28]. Hydrogenases were predicted and assigned to particular groups using the HydDB web tool [29]. Prediction and comparative analysis of the metabolic capabilities of MAGs was performed using the Distilled and Refined Annotation of Metabolism (DRAM) tool [30].

Genomic distances (average amino acid identity (AAI) and average nucleotide identity (ANI)) were calculated using tools from the Enveomics Collection [31].

### 2.5. Phylogenetic Analysis

Single-copy marker genes were identified, concatenated, and aligned using the GTDB-Tk v.0.3.2 tool. A maximum likelihood phylogenetic tree was constructed using PhyML v.3.3 [32] with default parameters. Branch support values were estimated using the Bayesian test in PhyML.

## 3. Results

### 3.1. Characteristics of the Sampling Site

The study site was an abandoned coal pit near the city of Gusinoozersk in Eastern Siberia. Three samples were collected and analyzed: Bu21 was a heated ground from a depth of 5–10 cm, Bu22 was a heated ground from a depth of 5–10 cm from a crack through which hot steam escaped, and Bu22-1 was sampled from a depth of 15–20 cm under the Bu22 sample and differed from it by a large number of sulfur inclusions. The ground temperature at the sampling sites ranged from 70 to 72 °C.

Electron microprobe analysis of rock samples showed high C and O content, which is to be attributed to the organic content of the lignite. Silicium and aluminium were abundant in most EDS microprobes, while the content of sulfur varied from 0.3% to 3.3% (Table 1). Inorganic minerals detected in the coal-included silicates were quartz (SiO_2_), muscovite (KAl₂[AlSi₃O₁₀](OH)₂), clinochlore (Mg_5_Al(AlSi_3_O_10_)(OH)_8_), albite (NaAlSi_3_O_8_), anorthite (CaAl_2_Si_2_O_8_), kaolinite (Al_2_(OH)_4_Si_2_O_5_), microcline (KAlSi_3_O_8_), and illite (2K_2_O_3_MgO Al_2_O_3_ 24SiO_2_ 12H_2_O). Sulfate minerals such as gypsum were not found; sulfur was present in amorphous and crystalline forms.

### 3.2. Microbial Community Compositions Revealed by 16S rRNA Gene Profiling

**Figure 2:**
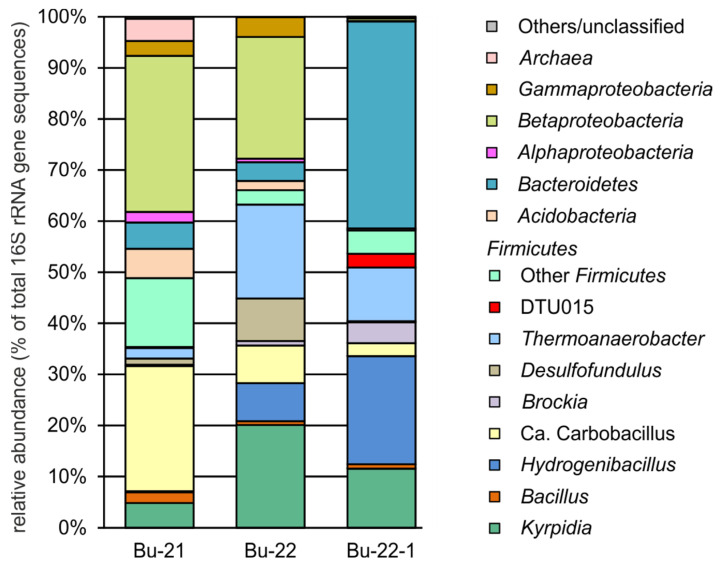
Microbial community composition revealed by 16S rRNA gene profiling.

A total of 153,354 high-quality 16S rRNA gene reads (53,048 for Bu21, 54,499 for Bu22 and 45,807 for Bu22-1) were used to analyze the microbial community composition. Prokaryotic communities were dominated by bacteria; archaeal 16S rRNA gene sequences were revealed only in sample Bu21 and accounted for about 4.35% of the total. The results of the taxonomic classification of the OTUs are outlined in Figure 2 and shown in detail in the Supplemental Table S1.

The archaeal population found in the Bu21 sample was represented by members of ANME-2a-2b (2.67% of all 16S rRNA gene reads), Marine Benthic Group D (0.09%), and *Nitrosopumilaceae* (1.59%). The closest members of ANME-2a-2b and MBGD have been found in various marine sediments. The family *Nitrosopumilaceae* was represented by two OTUs related to aerobic ammonia-oxidizing archaea *Nitrosopumilus adriaticus* (95.75% identity) and *Nitrosopumilus oxyclinae* (97.41% identity).

Most of the community in all three samples was represented by the phylum *Firmicutes*. The microbiome of sample Bu21 was dominated by *Candidatus* Carbobacillus altaicus (24.48%), a facultatively anaerobic heterotroph that can use molecular hydrogen as an energy source, previously described from the metagenome of burning coal sites [8]. In other samples its relative abundances were 7.36% (Bu22) and 2.57% (Bu22-1). Samples Bu22 and Bu22-1 were dominated by *Kyrpidia tusciae* (20.11 and 11.52%, respectively), an aerobic facultatively chemolitoautotrophic thermophilic bacterium oxidizing molecular hydrogen [33,34], and members of the family *Thermoanaerobacteraceae* (18.77 and 12.61%, respectively). The latter are anaerobes that can grow organoheterotrophically by fermentation or anaerobic respiration with sulfur and thiosulfate. Some species, such as *Thermoanaerobacter kivui*, can grow chemolithoautotrophically using molecular hydrogen as an electron donor and performing CO_2_ fixation in the Wood–Ljungdahl pathway [35].

In the Bu22-1 sample the most abundant phylotype among *Firmucutes* was *Hydrogenibacillus schlegelii* (21.13%)—an aerobic facultatively chemolitoautotrophic hydrogen-oxidizing bacterium [36]. This species was present in two other samples but in smaller amounts. Hydrogenotrophic *Firmicutes* were also represented by the anaerobic chemolithoautotrophic species *Brockia lithotrophica*, which was most abundant in the Bu22-1 sample (4.06%).

About 2% of 16S rRNA sequences in the Bu21 and about 1% in two other samples belonged to *Bacillus methanolicus* (98.3% 16S rRNA sequence identity to *B. methanolicus* MGA3), an aerobic thermophilic bacterium that uses methanol as its sole source of carbon and energy [37].

About 40% of the microbiome of the Bu22-1 sample was represented by a single OTU assigned to the family *Chitinophagaceae* (phylum *Bacteroidetes*). This OTU has 98.05% sequence identity with *Thermoflavifilum aggregans*, a thermophilic strictly aerobic heterotroph isolated from geothermally heated soil at Waikite, New Zealand [38].

*Betaproteobacteria* (the genera *Bordetella* and *Burkholderia*) were abundant in samples Bu21 (30.5%) and Bu22 (23.4%). Some members of these genera are known as human pathogens, but others occur in various natural habitats, including soils [39,40].

### 3.3. Metagenome Sequencing and Assembly of MAGs

Sequencing of the metagenome of the sample Bu22-1 using Illumina HiSeq2500 and Oxford Nanopore MinION platforms allowed us to recover 40 MAGs with more than 80% completeness and less than 10% contamination, as estimated by CheckM (Supplementary Table S2). In total, these MAGs accounted for about 62% of all metagenome sequences.

Taxonomic assignment of the MAGs based on the searches against GTDB revealed the same major bacterial lineages that were found according to the 16S rRNA gene profiling. To characterize the metabolic potential of the microbial lineages that were numerous in the community and/or involved in the oxidation of coal gases, we analyzed some obtained MAGs.

### 3.4. Firmicutes

The dominant phylum, *Firmicutes*, accounted for 56% of the metagenome and was represented by 27 MAGs (Supplementary Table S2). The most abundant *Firmicutes* lineage, defined as an order *Thermicanales* in the GTDB R95 taxonomy, comprised five MAGs and accumulated 40% of the metagenome. The major genotype, MAG Bu28, accounting for 32.7% of the metagenome, was classified as *Hydrogenibacillus schlegelii*, with 95.73% AAI to *H. schlegelii* strain MA48. Another less abundant MAG, Bu13, shared 70.41% AAI with *H. schlegelii* MA48 and probably represented another species of this genus.

Two less numerous genotypes, Bu53 and Bu36, were assigned to Ca. Carbobacillus altaicus (97.85% AAI with Ca. Carbobacillus altaicus AL32) and probably a new *Thermicanus* species (93.37% AAI with the only cultured member of this genus, *Thermicanus aegyptius*), respectively. Genome analysis of Ca. Carbobacillus altaicus Bu53, two *Hydrogenibacillus* genotypes (Bu28 and Bu13), and *Thermicanus* Bu36 predicted that these bacteria are capable of aerobic hydrogen oxidation, like previously described members of these genera [8,36,41].

MAG Bu66 shared 99.43% AAI with *Brockia lithotrophica* Kam1851and was assigned to this species. *B. lithotrophica* was first isolated from a hot spring in Kamchatka, Russia [42]. It is an obligately anaerobic spore-forming chemolithoautotrophic thermophile using molecular hydrogen or formate as electron donors and elemental sulfur, thiosulfate, or polysulfide as electron acceptors [42]. Genes for the [NiFe] group 1d uptake hydrogenase and formate dehydrogenase were identified in the Bu66 genome. The reduction of sulfur and/or thiosulfate could be performed by the complex iron–sulfur molybdoenzyme (CISM) oxidoreductase of the Psr/Phs family, including the genes of molybdopterin-binding catalytic subunit A, related to thiosulfate and polysulfide reductases, the iron-sulfur electron transfer subunit B, and membrane-linking subunit C.

Analysis of the possible pathways of autotrophic carbon fixation revealed the presence of the Calvin cycle in *H. schlegelii* Bu28, *Hydrogenibacillus* Bu13, and *B. lithotrophica* Bu66. The key enzyme of this pathway, ribulose 1,5-bisphosphate carboxylase (RuBisCO), in all these bacteria belongs to type 1-E [43]. Ca. Carbobacillus altaicus Bu53 and *Thermicanus* Bu36 lacked the Wood–Ljungdahl pathway, the Calvin cycle, the hydroxypropionate-hydroxybutylate cycle, and the reverse tricarboxylic acid cycle, indicating that these bacteria are probably devoted to a heterotrophic lifestyle.

All *Thermicanales* MAGs contained membrane-bound [NiFe] -family uptake hydrogenases that could provide an electron input for aerobic and/or anaerobic respiration [44]. *Hydrogenibacillus* Bu13 additionally contained aerobic carbon monoxide dehydrogenase, which can enable the use of CO as an energy source. CO dehydrogenase was also detected in the *H. schlegelii* MA48 genome, although its growth on CO has not been tested [36], while the *H. schlegelii* Bu28 MAG lacked this gene cluster. CO dehydrogenase of an anaerobic type was found in *B. lithotrophica* Bu66, although it is absent in the type strain *B. lithotrophica* Kam1851.

Another group of thermophilic hydrogenotrophic *Firmicutes*, the family *Kyrpidiaceae* recognized in the GTDB taxonomy, comprised three MAGs and accounted for 3.26% of the metagenome. The most abundant genotype, MAG Bu76, shared 97.45% AAI with *Kyrpidia tusciae* DSM 2912 and likely belongs to this species. *Kyrpidia tuscia* is a thermophilic, hydrogen-oxidizing chemolithoautotroph. Another *Kyrpidia* species, *Kyrpidia spormannii*, can grow autotrophically by oxidizing H_2_ and CO [45]. The genes encoding RuBisCo, group 2a [NiFe] type uptake hydrogenase, CO dehydrogenase, and the aerobic respiratory chain were found in the Bu76 genome, which indicates that this bacterium can use coal gases as energy sources. The presence of gene clusters encoding a multisubunit aromatic/alkene monooxygenase hydroxylase family enzyme indicates that the Bu76 bacterium could probably oxidize low molecular weight hydrocarbons known to occur in coal gases.

The most abundant anaerobic group of *Firmicutes*, the family *Thermoanaerobacteraceae*, accounted for 5.1% of the metagenome and was dominated by MAG Bu09, closely related (91.18% AAI) to *Thermoanaerobacter* sp. AS04akNAM_88 [46]. The Bu09 bacterium lacked autotrophic carbon fixation pathways and could probably grow by the fermentation of carbohydrates as suggested by the presence of numerous glycoside hydrolases. The presence of anaerobic CO dehydrogenase in MAG Bu09 suggests that this bacterium could use CO from coal gases as an energy source.

Among known sulfate-reducing *Firmicutes* lineages, members of the families *Desulfovirgulaceae* and *Moorellaceae* were identified. The most abundant genotype, MAG Bu69 (1.1% of the metagenome), shared 94.8% AAI with *Desulfofundulus australicum* DSM 11792. This sulfate-reducing bacterium can utilize CO both in the presence and in the absence of sulfate and dominates in syngas-degrading enrichment cultures [47]. Analysis of the Bu69 genome revealed CO dehydrogenase, the Wood–Ljungdahl pathway for carbon fixation, and all genes required for dissimilatory sulfate reduction, thus suggesting that this organism is likely devoted to a similar lifestyle.

The main properties of the above-described MAGs are summarized in Table 2.

### 3.5. Bacteroidetes

*Bacteroidetes* were the second most abundant lineage in the Bu22-1 sample and were represented by a single genotype both in 16S rRNA gene library and in the metagenome. Interestingly, this MAG (Bu77) comprised only 3.2% of the metagenome, while the corresponding OTU2 accumulated about 40% of 16S rRNA gene reads. This MAG was consistently assigned to the genus *Thermoflavfilum*, with 83.1% AAI between this genome and its closest relative, *Thermoflavifilum aggregans*. *T. aggregans* is an aerobic organotroph with a temperature optimum of about 60 °C, isolated from geothermally heated soil at Waikite, New Zealand [38]. Apparently, the Bu77 bacterium uses various carbohydrates as substrates, as evidenced by the presence of a large number of genes of various glycosyl hydrolases. Genes for respiratory hydrogenases and CO dehydrogenases were not identified. Analysis of the Bu77 genome revealed complete pathways of glycolysis and gluconeogenesis, the pentose phosphate pathway including both oxidative and non-oxidative stages, and the tricarboxylic acid cycle. A complete aerobic respiratory chain is present with a terminal cytochrome c oxidase. The known autotrophic carbon fixation pathways, in particular, the Wood–Ljungdahl pathway and the Calvin cycle are missing, and the absence of citrate lyase indicates that the operation of TCA in reverse for carbon fixation is unlikely. Therefore, *Thermoflavfilum* Bu77 is probably an aerobic heterotroph that can mineralize organics produced by other community members but is unable to use coal gases as an energy source.

### 3.6. Bacterium Bu02 of Uncultured Division DTU015

Taxonomic assignment of one of the *Firmicutes* MAGs, Bu02, placed it into the class-level uncultured division DTU015 in the phylum Firmicutes_E in the GTDB taxonomy. This genome was obtained as a single circular contig with a length of 2,316,832 bp. The relative abundance of Bu02 in the metagenome was about 1%, a value consistent with a 1.6% share of the corresponding OTU21 in the 16S rRNA gene pool.

Taking into account that we obtained the first complete genome for the candidate class DTU015, the phylogenetic position and metabolic potential of the Bu02 bacterium were analyzed in detail. As a result of genome annotation, 2147 potential protein-coding genes were predicted, and the functions of only half of them were tentatively assigned. Two copies of the rRNA operon (16S-23S-5S) and 46 tRNA genes were identified.

The presence of genes encoding the rod-shaped determining proteins MreBCD, RodA, and peptidoglycan D,D-transpeptidase MrdA [48] suggests that cells of the Bu02 bacterium are rod-shaped. The discovery of genes for flagellar machinery and chemotaxis indicates that the Bu02 bacterium is probably motile. It is likely able to form endospores, as indicated by the presence of a set of genes required for sporulation. A notable feature is the presence of a *gvp* gene cluster coding for the gas vesicles that could confer buoyancy for cells [49].

The Bu02 16S rRNA gene shares only 86% sequence identity with the closest cultured bacterium, *Desulfofundulus solfataricus*. Considering uncultured organisms, a GeneBank search revealed the closest 16S rRNA sequence (AY526501, 99.6% identity) from thermophilic anaerobic sludge [50]. The search for 16S rRNA sequences with >95% identity to Bu02 in the Joint Genome Institute Integrated Microbial Genomes database (16S rRNA Public Assembled Metagenomes) revealed a total of 26 sequences (>700 bp) presumably representing members of the same genus. Twenty-one clones have been identified in anaerobic digesters and five clones in sediments of hot springs from Yellowstone National Park. Consistently, GTDB searches revealed a close relative of Bu02 (MAG AS04akNAM_21 with 96.73% AAI) in an anaerobic thermal digester [51].

**Figure 3:**
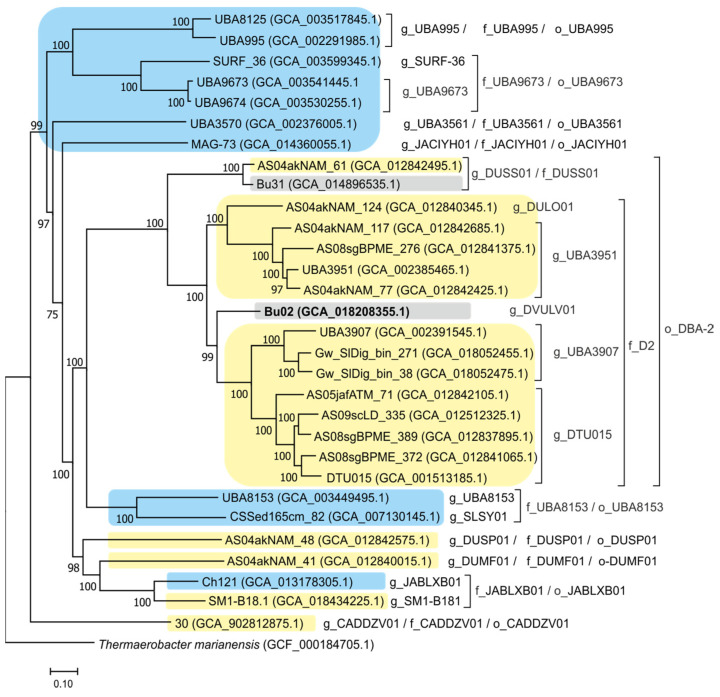
Phylogenomic placement of the Bu02 genome in the maximum likelihood concatenated protein phylogeny of class DTU015. The level of support for internal branches was assessed using the Bayesian test in PhyML. Taxonomy is shown according to the GTDB release R207 (c, class; o, order; f, family). The genome of *Thermaerobacter marianensis* representing a sister class *Thermaerobacteria* of the candidate phylum Firmicutes_E was used to root the tree. Genomes from natural aquatic environments are marked in blue, those from anaerobic digesters and wastewater are marked in yellow, and ones from coal-fire sites are marked in grey.

To characterize the phylogenetic position of the Bu02 bacterium, a phylogenetic tree based on concatenated sequences of the conserved genes of bacteria assigned to the phylum Firmicutes_E in the GTDB taxonomic system was constructed. The obtained tree confirmed that Bu02 belongs to class DTU015 and placed it within the candidate order D8A-2 and family D2, as defined by the GTDB taxonomy (Figure 3).

**Figure 4:**
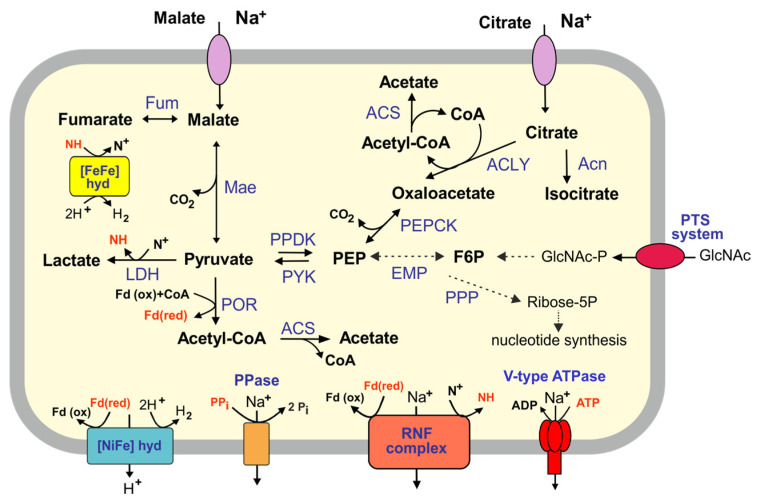
An overview of metabolic pathways encoded in the Bu02 genome. Abbreviations: EMP, Embden-Meyerhof (EM) pathway; PPP, pentose-phosphate pathway; PYK, pyruvate kinase; PPDK, pyruvate phosphate dikinase; POR, pyruvate ferredoxin oxidoreductase; LDH, lactate dehydrogenase; ACS, acetyl-CoA synthetase (ADP-forming); Mae, malic enzyme; PEPCK, phosphoenolpyruvate carboxykinase; ACLY, citrate lyase; Acn, aconitase; Fum, fumarate hydratase; hyd, hydrogenase; PPase, pyrophosphatase; Fd(ox)/Fd(red), ferredoxin, oxidized and reduced form; N^+^, NAD(P)^+^; NH, NAD(P)H; CoA, coenzyme A.

Analysis of the Bu02 genome revealed the presence of all genes of the Embden–Meyerhof (EM) pathway except for glucokinase and glucose-6-phosphate isomerase, performing first two steps of glycolysis. The presence of phosphoenolpyruvate synthase and fructose 1,6-bisphosphatase in the absence of glucose 6-phosphatase indicates that gluconeogenesis might proceed up to fructose 6-phosphate. Pyruvate generated in the EM pathway may be decarboxylated to acetyl-coenzyme A (CoA) by pyruvate: feredoxin oxidoreductase or converted into lactate by lactate dehydrogenase. Acetyl-CoA synthetase (ADP-forming) probably catalyzes the formation of acetate from acetyl-CoA with the concomitant generation of ATP. The re-oxidation of reduced ferredoxin generated by pyruvate: ferredoxin oxidoreductase might be performed by hydrogenase, which reduces protons to H_2_ (Figure 4).

The search for potential hydrolytic enzymes revealed a very limited range of glycoside hydrolases and carbohydrate transporters. Therefore and consistently with the absence of two upper glycolytic enzymes, it is unlikely that the Bu02 bacterium is specialized on the utilization of sugars. The only apparent exception is *N*-acetylglucosamine (GlcNAc) and corresponding oligosaccharides. Genome analysis revealed GH18 family chitinase, beta-*N*-acetylglucosaminidase of the GH3 family, and a mannose/fructose/*N*-acetylgalactosamine-specific phosphotransferase system that could enable the utilization of these substrates [52]. Upon phosphorylation and import into the cytoplasm, the GlcNAc-6-P might be converted into fructose-6-P via the action of an *N*-acetylglucosamine-6-phosphate deacetylase and glucosamine-6-phosphate deaminase and enter the glycolytic pathway.

The Bu02 genome encodes several transport systems for an uptake of amino acids and short peptides, namely four oligopeptide uptake ABC transporters of the Peptide/Opine/Nickel Uptake Transporter Family (TC 3.A.1.5), di- and tri-peptide ABC transporter (TC 3.A.1.5.39), and ABC transporter of the Polar Amino Acid Uptake Transporter Family (TC 3.A.1.3). The presence of intracellular peptidases, transaminases, NADP-specific glutamate dehydrogenase, 2-oxoacid ferredoxin oxidoreductases (pyruvate ferredoxin oxidoreductase, two 2-oxoglutarate/2-oxoacid ferredoxin oxidoreductases, and indolepyruvate oxidoreductase), and other enzymes involved in amino acid degradation suggests that the Bu21-1-02 bacterium could import and ferment short peptides and amino acids.

Enzymes of the nonoxidative stage of the pentose phosphate pathway, ribulose phosphate 3-epimerase, ribose-5-phosphate isomerase, transketolase, and transaldolase, are encoded. Ribose-5-phosphate might be phosphorylated by ribose-phosphate pyrophosphokinase to make phosphoribosyl pyrophosphate, a key intermediate for nucleotide biosynthesis. Consistently, the Bu02 genome contained a set of genes for the *de novo* biosynthesis of nucleotides.

Analysis of the Bu02 genome revealed the absence of genes of the TCA cycle, except for aconitate hydratase and fumarate hydratase. However, the presence of transporters of the 2-Hydroxycarboxylate Transporter (2-HCT) Family (TC 2.A.24) that catalyze citrate or malate uptake with either Na^+^ or H^+^ as the co-transported cation [53] suggest that these TCA intermediates could be used as substrates. Citrate could be converted to oxaloacetate and acetyl-CoA by ATP citrate lyase, while NADP-dependent malic enzyme could decarboxylate malate to pyruvate. Oxaloacetate could be converted into phosphoenolpyruvate and carbon dioxide by the action of phosphoenolpyruvate carboxykinase (Figure 4).

The components of electron transfer chains, NADH dehydrogenase, membrane-bound succinate dehydrogenase/fumarate reductase, cytochrome *bc_1_* complex, and terminal reductases for aerobic and anaerobic respiration processes, were not found, nor was F_0_F_1_-type H^+^-transporting ATPase, indicating that Bu02 is limited to fermentative metabolism.

The Bu02 bacterium has a number of mechanisms to generate a transmembrane ion gradient that can be used for transport purposes and motility. The genome contained a gene cluster encoding a multisubunit [NiFe] group 4g hydrogenase. Some of its subunits have transmembrane domains, so the hydrogenase is likely to be membrane-bound. Such enzymes can couple ferredoxin oxidation to proton reduction and translocate sodium or protons out of the cytoplasm [44]. The Bu02 genome also contains genes *rnfCDEAB* for the ion-transporting complex Rnf. In *Firmicutes* this membrane-linked complex acts catabolically, as ferredoxin: NAD^+^ oxidoreductase moving sodium ions or protons across the membrane out of the cell [54]. In addition, the genome encodes V-type ATPase, which uses ATP hydrolysis for the transportation, and a pyrophosphate-energized ion pump. The presence of the sodium-binding motif in the K subunit of V-type ATPase [55] and the GNXXA motif in pyrophosphatase [56] suggests that these enzymes transport sodium ions rather than protons.

**Figure 5:**
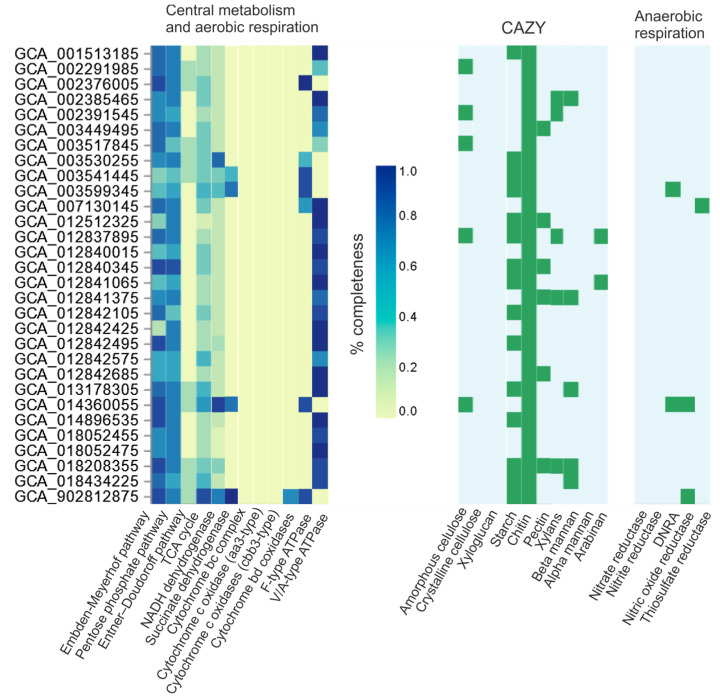
Comparative genome analysis of the members of analysis of the candidate class DTU015 performed using the DRAM tool. CAZY, carbohydrate-active enzymes (the presence of enzymes capable of hydrolyzing the substrates indicated at the bottom); DNRA, cytochrome c nitrite reductase (ammonia-forming).

At present, the candidate class DTU015 includes 30 species represented by the corresponding MAGs (Figure 3). In order to obtain information about the lifestyle of class DTU015 bacteria, we conducted a comparative genomic analysis of its representatives and analyzed the presence of important metabolic pathways using the DRAM tool (Figure 5). Since only the Bu02 genome is complete, the absence or incompleteness of particular genes/pathways in other MAGs should be judged with caution as it may result from the incompleteness of the assembly.

All members of DTU015 seem to have most of the genes of Embden–Meyerhof pathway and the nonoxidative stage of the pentose phosphate pathway. Cytochrome *c* oxidases were absent in all DTU015 genomes, while cytochrome *bd* oxidase was found only in a single MAG (GCA_902812875.1) representing a basal lineage of this class (o_CADDZV01). This MAG also encodes a TCA cycle, a respiratory chain with NADH dehydrogenase, succinate dehydrogenase, and F_0_F_1_-type ATPase, and nitric oxide reductase, indicating the respiratory capacity of this bacterium. Besides this MAG, genes encoding terminal reductases of anaerobic respiration were identified in only three genomes: one MAG (GCA_014360055.1) contained nitrite reductase *nrfA* and nitric oxide reductase, one MAG (GCA_012840345.1) *nrfA*, and another MAG thiosulfate/polysulfide reductase. Other members of DTU015 seem to be devoted to only a fermentative lifestyle.

The Bu02 MAG is the first complete genome in the candidate class DTU015 of the *Firmicutes*, recognized in the GTDB taxonomy. Since it is a finished MAG [57], taking into account the consensus statement on the naming of uncultivated prokaryotes [58], we propose the following taxonomic names.

Description of “*Candidatus* Fermentithermobacillus” gen. nov. Fermentithermobacillus (Fer.men.ti.ther’mo.ba.cil’lus. L. neut. gen. n. fermenti, of a fermentation process; Gr. adj. thermos, warm; L. masc. n. bacillus, a rod; a thermophilic rod-shaped bacterium performing fermentation processes).

Description of “*Candidatus* Fermentithermobacillus carboniphilus” sp. nov. Fermentithermobacillus carboniphilus (car.bo.ni’phi.1us. L. masc. n. carbo, coal; Gr. adj. philos, loving; M. L. adj. carboniphilus, carbon loving).

Not cultivated. Inferred to be an anaerobic thermophile, obligate organotroph fermenting low-molecular-weight organic substrates, including *N*-acetylglucosamine, peptides and amino acids. Cells were predicted to be rod-shaped and capable of flagellar motility. Represented by the complete genome assembled from the metagenome of the rocky soil sample heated by an underground coal fire near the town of Gusinoozersk, Republic of Buryatia, Russian Federation.

Based on this, we propose the following names for the class, order and family:

“Candidatus Fermentithermobacillia” classis nov.

“Candidatus Fermentithermobacillales” ord. nov.

“Candidatus Fermentithermobacillaceae” fam. nov.

## 4. Discussion

In this work, we investigated the microbial communities of the near-surface ground layer in an open coal mine, associated with gas vents from an underground coal fire. The fire manifested itself in the form of intense heating of the ground and the rise of steam and smoke. Analysis of the composition of the microbial communities of three ground samples both by 16S rRNA gene profiling and by metagenomic analysis revealed a rather simple community dominated by only a few groups of microorganisms. Most of them are known thermophilic lineages of the phylum *Firmicutes*, both aerobic and anaerobic ones. Although the samples were collected at different depths (5–10 cm and 15–20 cm), we did not observe the clear prevalence of anaerobic lineages (e.g., *Thermoanaerobacter* sp.) in the deeper versus the upper ground layer. Since the collected samples were finely dispersed coal-bearing rock particles, we suppose that local anaerobic zones were formed within the ground particles while oxygen was available at all sampling depths.

Hydrogenotrophy is a common metabolic feature of *Firmicutes* that dominate microbial communities associated with coal gas vents. Ca. Carbobacillus, *Kyrpidia*, *Hydrogenibacillus*, and *Thermicanus* can oxidize hydrogen under aerobic conditions. *Brockia* is capable of anaerobic respiration with sulfur compounds. The ability to oxidize molecular hydrogen was based on the presence in the relevant MAGs of [NiFe] group 1d and/or [NiFe] group 2a hydrogenases, known to be involved in hydrogenotrophic respiration [59]. Another high-energy component of the coal gases, carbon monoxide, can be used by one of two *Hydrogenibacillus* phylotypes, *Kyrpidia*, and *Brockia* (Table 2). Consistently with the predicted lifestyle, these MAGs encoded either aerobic (*Hydrogenibacillus*, *Kyrpidia*) or anaerobic (*Brockia*) types of respiratory CO dehydrogenase.

Other simple organic compounds generated in reactions involving coal gases could be used by some community members. Methanol that could be produced from syngas components in the reaction CO + 2H_2_ → CH_3_OH could support the growth of *Bacillus methanolicus*, an aerobic thermophilic methanol oxidizer. *Kyrpidia tusciae* Bu76 was predicted to utilize alkenes and other low molecular weight hydrocarbons known to be present in coal gases [1,2].

Sulfur compounds present in coal seams can be carried to the surface with a gas stream. Consistently, the presence of sulfur was revealed by electron microprobe analysis. The Bu22-1 sample was particularly rich in sulfur and contained easily visible sulfur deposits. Most numerous bacterial lineages, *H. schlegelii* Bu28, *B. lithotrophica* Bu66, and *K. tusciae* Bu76, possessed genes for membrane-linked CISM oxidoreductase of the Psr/Phs family, known to have activities of polysulfide, thiosulfate, and tetrathionate reductases [60]. Therefore, respiration with these sulfur compounds could be an important process in an anaerobic zone. On the contrary, sulfate reducers were not abundant in the community, and sulfate-containing minerals were not identified in the ground samples.

MAGs assigned to *Hydrogenibacillus*, *Brockia*, and *Kyrpidia* contain all enzymes of the Calvin cycle enabling autotrophic carbon fixation, including type 1-E RuBisCO. This type of RuBisCO, along with high affinity group 1h [NiFe] uptake hydrogenases and aerobic carbon monoxide dehydrogenases, was recently found in metagenomes of microbial communities in oligotrophic cold desert soils, and it was proposed that H_2_ and CO found in the atmosphere at the trace concentrations, and CO_2_ provide sources of energy and carbon to support these communities in a process called atmospheric chemosynthesis [61,62]. We suppose that coal gas vents provide much higher local concentrations of H_2_ than atmospheric levels, thus enabling the hydrogenotrophic growth of organisms with more widespread uptake hydrogenases of types 1d and 2a.

Interestingly, very close relatives of the hydrogenotrophic *Firmicutes* found in this work were previously detected in a coal fire site in the Altai Mountains [8]. *B. lithotrophica* Bu66, Ca. Carbobacillus altaicus Bu53, and *H. schlegelii* Bu28 shared 99.48%, 98.96%, and 96.34% ANI with MAGs AL31, AL32, and AL33 from the Altai Mountains site, respectively. Several strains of *H. schlegelii* were isolated from Alpine lake sediments [36], geothermally heated soils in Antarctica [63], and even air [64]. The only cultured isolate of *B. lithotrophica* was obtained from a terrestrial hot spring of the Uzon Caldera, Kamchatka Peninsula, Russia [42], and several closely related (>97% identity) 16S rRNA gene sequences were retrieved from Wilbur Hot Springs, California, USA. *Kyrpidia tusciae* was isolated from the solfatara of San Federigo, a geothermal area in Tuscany, Italy [33]. Another *Kyrpidia* species, *K. spormannii*, was obtained from hydrothermal sediment samples from the Azores, Portugal [65] and from the geothermal soils on Pantelleria Island, Italy [45]. 16S rRNA gene profiling showed that sequences closely related to *Kyrpidia tusciae* Bu76 also occurred in non-geothermal environments characterized by elevated temperatures, such as sugarcane bagasse feedstock piles with a temperature of 49–52 °C [66] and thermophilic microbial fuel cells inoculated with biomass from a methanogenic anaerobic digester [67]. A search for 16S rRNA sequences related to Ca. Carbobacillus altaicus Bu53 revealed several clones from thermophilic microbial fuel cells [67] and leachate microbial communities from a municipal landfill in Southern Ontario, Canada, with 95–96% identity.

Endospores of *Firmicutes* can survive multiple environmental stress conditions, such as ionizing radiation, heat, pressure, desiccation, pH extremes, and toxic chemicals. Such resistance facilitates the global dispersion of these organisms. For example, spore-forming thermophilic *Desulfotomaculum* sp. originating from geothermal sites have been isolated from sub-seafloor sediments [68] where their spores have been shown to have a half-life of up to ∼300 years [69]. It was suggested that the vegetative life of *H. schlegelii* occurs in geothermal sites while the transport of its spores can occur by wind dispersal [64]. Therefore, it seems likely that spores of thermophilic *Firmicutes* can spread over long distances, which allows these microorganisms to colonize newly formed thermal ecological niches. The fast global dispersion of these bacteria could explain high genome similarity between hydrogenotrophic *Firmicutes* found in this work and the same species detected in the coal fire site in the Altai Mountains, located at about 1300 km distance.

The ability to form spores may also be an important factor for the survival of bacteria at a particular coal fire site. The exit points of coal gases often move within the coal combustion site even in the course of one year, and therefore it is important for the bacterium to survive a temporary drop in temperature and a lack of substrates.

This study provides the first insight into the biology of a member of the candidate class DTU015 of the phylum Firmicutes_E recognized in the GTDB taxonomy. In contrast to hydrogenotrophic *Firmicutes*, which constituted the majority of microbial communities, Bu02 is a fermentative bacterium lacking an aerobic and anaerobic respiratory chain. The Bu02 bacterium was predicted to be specialized in the utilization of low molecular weight organic substrates, including peptides, amino acids, intermediates of the TCA cycle (citrate and/or malate), and a limited set of simple sugars (e.g., *N*-acetylglucosamine). Close relatives of Bu02 were detected by 16S rRNA gene profiling in thermal anaerobic digesters, while somewhat more distant relatives were detected in sediments of hot springs. Comparative genomic analysis showed that a fermentative anaerobic lifestyle is characteristic of nearly all members of DTU015, and therefore we proposed the name “*Candidatus* Fermentithermobacillia” for this class. In the studied ecosystem, Bu02 occupying an anaerobic zone probably plays the role of scavenger, carrying out the fermentation of low molecular weight organic substances formed by autotrophic *Firmicutes*.

## 5. Conclusions

Microbial communities of the near-surface ground layer of an open quarry heated by a subsurface coal fire were dominated by only a few groups of spore-forming *Firmicutes*, namely the aerobic heterotroph *Candidatus* Carbobacillus altaicus, the aerobic chemolitoautotrophs *Kyrpidia tusciae* and *Hydrogenibacillus schlegelii*, and the anaerobic chemolithoautotroph *Brockia lithotrophica*. Genome analysis predicted that these species can obtain energy from the oxidation of hydrogen and/or carbon monoxide in coal gases. Very close relatives of hydrogenotrophic *Firmicutes* found in this work were previously detected in geographically distant thermal sites. Considering the predicted ability of all these species to form spores that could spread over long distances, such high genome similarity could be explained by the fast global dispersion of these bacteria.

We assembled the first complete closed genome of a member of uncultured class-level division DTU015 in the phylum *Firmicutes*. This bacterium, “*Candidatus* Fermentithermobacillus carboniphilus” Bu02, was predicted to be rod-shaped and capable of flagellar motility and sporulation. Genome analysis showed the absence of aerobic and anaerobic respiration and suggested a facultatively anaerobic chemoheterotrophic lifestyle with the ability to ferment peptides, amino acids, *N*-acetylglucosamine, and tricarboxylic acid cycle intermediates. The Bu02 bacterium probably plays the role of a scavenger, performing the fermentation of organics formed by autotrophic *Firmicutes* supported by coal gases. A comparative genome analysis of the DTU015 division revealed that the vast majority of its members have a similar lifestyle. Based on genome data, we propose the following names for the class, order, and family: “Candidatus Fermentithermobacillia” classis nov., “Candidatus Fermentithermobacillales” ord. nov., and “Candidatus Fermentithermobacillaceae” fam. nov.

## Figures and Tables

**Table 1:** The average atomic percentages of the elements calculated from five microprobes.

Element	Atomic Percentage ^1^				
	1	2	3	4	5
C	46.78	58.65	69.17	51.11	44.82
O	37.60	26.39	22.65	33.26	36.00
Na	0.06	0.14	0.12	0.21	1.37
Mg	0.41	0.30	0.30	0.41	0.53
Al	5.04	3.30	1.77	3.97	4.82
Si	8.16	6.04	2.82	9.23	9.81
S	0.27	3.31	1.76	0.30	0.27
K	0.65	0.51	0.33	0.51	0.97
Ca	0.16	0.25	0.47	0.13	0.35
Fe	0.87	1.10	0.61	0.86	1.06

^1^ Five analyzed microprobes.

**Table 2:** Genetic determinants of autotrophic carbon fixation and utilization of coal gases in *Firmicutes* MAGs.

MAG	Taxonomic Assignment	Psr/Psh Reductase	Cytochrome c Oxidase	CO Dehydrogenase	Respiratory [NiFe] Hydrogenase	Calvin Cycle	WLP ^1^
Bu28	*Hydrogenibacillus schlegelii*	+	+	-	Group 1d Group 2a	+	−
Bu13	*Hydrogenibacillus* sp.	−	+	aerobic	Group 2a	+	−
Bu53	*Ca.* Carbobacillus altaicus	−	+	-	Group 2a	−	−
Bu36	*Thermicanus* sp.	−	+	-	Group 1d	−	−
Bu66	*Brockia lithotrophica*	+	−	anaerobic	Group 1d	+	−
Bu76	*Kyrpidia tusciae*	+	+	aerobic	Group 2a	+	−
Bu09	*Thermoanaerobacter* sp.	−	−	anaerobic	-	−	−
Bu69	*Desulfofundulus* sp.	+	−	anaerobic	-	−	+
Bu02	DTU015	−	−	-	-	−	−

^1^ Wood–Ljungdahl pathway.

## Data Availability

The raw data generated from 16S rRNA gene profiling and metagenome sequencing, and MAG assemblies are accessible via the BioProject PRJNA663466 (Supplemental Table S3).

## Supplementary Materials

The following supporting information can be downloaded at: https://www.mdpi.com/article/10.3390/biology12050723/s1, Table S1: Relative abundance and taxonomic classification of OTUs; Table S2: Main characteristics of MAGs obtained in this study; Table S3: Nucleotide sequence accession numbers.
